# A combined radiomics and habitat analysis model for predicting early recurrence of HCC after liver transplantation

**DOI:** 10.3389/fonc.2026.1789990

**Published:** 2026-05-26

**Authors:** Ya Yang, Yong Luo, Hui Liu, Mengyi Wang, Xingwu Xiao, Li Zhang, Xiaolei Su, Bin Shi, Junming Wang, Hao Xie

**Affiliations:** 1 Department of Ultrasound Medicine, West China Hospital Sichuan University Jintang Hospital, Jintang First People’s Hospital, Chengdu, Sichuan, China; 2 Department of Radiology, West China Hospital Sichuan University Jintang Hospital, Jintang First People’s Hospital, Chengdu, Sichuan, China; 3 Postgraduate Training Base of the Third Medical Center of Chinese People's Liberation Army (PLA) General Hospital, Jinzhou Medical University, Beijing, China; 4 Department of Organ Transplantation, The Third Medical Center of People's Liberation Army (PLA) General Hospital, Beijing, China; 5 Department of Urology, West China Hospital Sichuan University Jintang Hospital, Jintang First People’s Hospital, Chengdu, Sichuan, China

**Keywords:** computed tomography, habitat analysis, hepatocellular carcinoma, liver transplantation, radiomics, recurrence

## Abstract

**Introduction:**

Hepatocellular carcinoma (HCC) is highly prevalent and ranks as the third leading cause of cancer-related deaths globally, posing a significant threat to public health worldwide. To identify high-risk patients for early recurrence after liver transplantation (LT) promptly, thereby optimizing personalized follow-up and intervention strategies for HCC patients. We aim to develop a combined radiomics and habitat analysis model for predicting early recurrence of HCC After LT.

**Methods:**

A retrospective cohort of 140 HCC patients was selected. Arterial-phase CECT images and clinical data were used for region of interest (ROI) segmentation, habitat analysis, feature extraction and selection. Model performance was assessed through receiver operating characteristic (ROC) curves, calibration curves, and decision curve analysis (DCA).

**Results:**

The accuracy, sensitivity, specificity, and AUC values of the combined model in the training and testing cohorts were 0.847/0.833, 0.830/0.731, 0.867/1.000, and 0.929 (95% CI: 0.883–0.975)/0.882 (95% CI: 0.983–0.982), all outperforming the clinical model, radiomics model, and habitat analysis model. Furthermore, the combined model demonstrated the highest consistency in calibration curves and provided the greatest clinical net benefit in decision curve analysis.

**Conclusion:**

The combined model based on radiomics and habitat analysis demonstrates robust applicability and high predictive performance in identifying early postoperative recurrence of HCC. This strategy facilitates proactive and individualized intervention for high-risk patients, thereby improving clinical outcomes.

## Introduction

Hepatocellular carcinoma (HCC) is highly prevalent and ranks as the third leading cause ofcancer-related deaths globally, posing a significant threat to public health worldwide (1).. Surgical resection is the preferred treatment for HCC, but studies show that about 70% of patients experience recurrence within five years (2–4). Liver transplantation (LT) is a highly effective treatment for HCC, but recurrence rates peak within 2–3 years post-transplantation (5). Early recurrence, defined as recurrence within the first year after transplantation, is linked to a poor prognosis (6). Research consistently shows that early recurrence in patients leads to significantly poorer outcomes than late recurrence (7). Consequently, accurately evaluating a patient’s risk of early recurrence is essential for informed clinical decision-making and the development of personalized treatment strategies (8).

Radiomics has recently gained attention as a promising non-invasive, high-throughput imaging technology for tumor diagnosis, treatment, and prognosis (9). Traditional radiomics research often assumes that tumors display heterogeneity with well-integrated components. However, solid cancers are characterized by significant spatial and temporal heterogeneity (10), featuring distinct cellular populations and spatial distributions within the tumor (11). Previous research has often neglected the local phenotypic variations within tumors (12). Habitat analysis, an innovative radiomics strategy that utilizes radiographic imaging, addresses this gap by describing the heterogeneity of each voxel within a tumor region. A voxel is a volume element and represents the smallest imaging unit in the three-dimensional space of a CT image. It employs clustering methods to group similar voxels, drawing parallels to ecological habitat analysis used in biodiversity studies (11, 13). Consequently, habitat analysis offers valuable insights for comprehensively quantifying intratumoral heterogeneity (ITH) and evaluating performance across distinct tumor subregions. This method has been utilized to evaluate chemotherapy responses in breast cancer (14) patients and esophageal cancer (15) patients.

**Figure 1:**
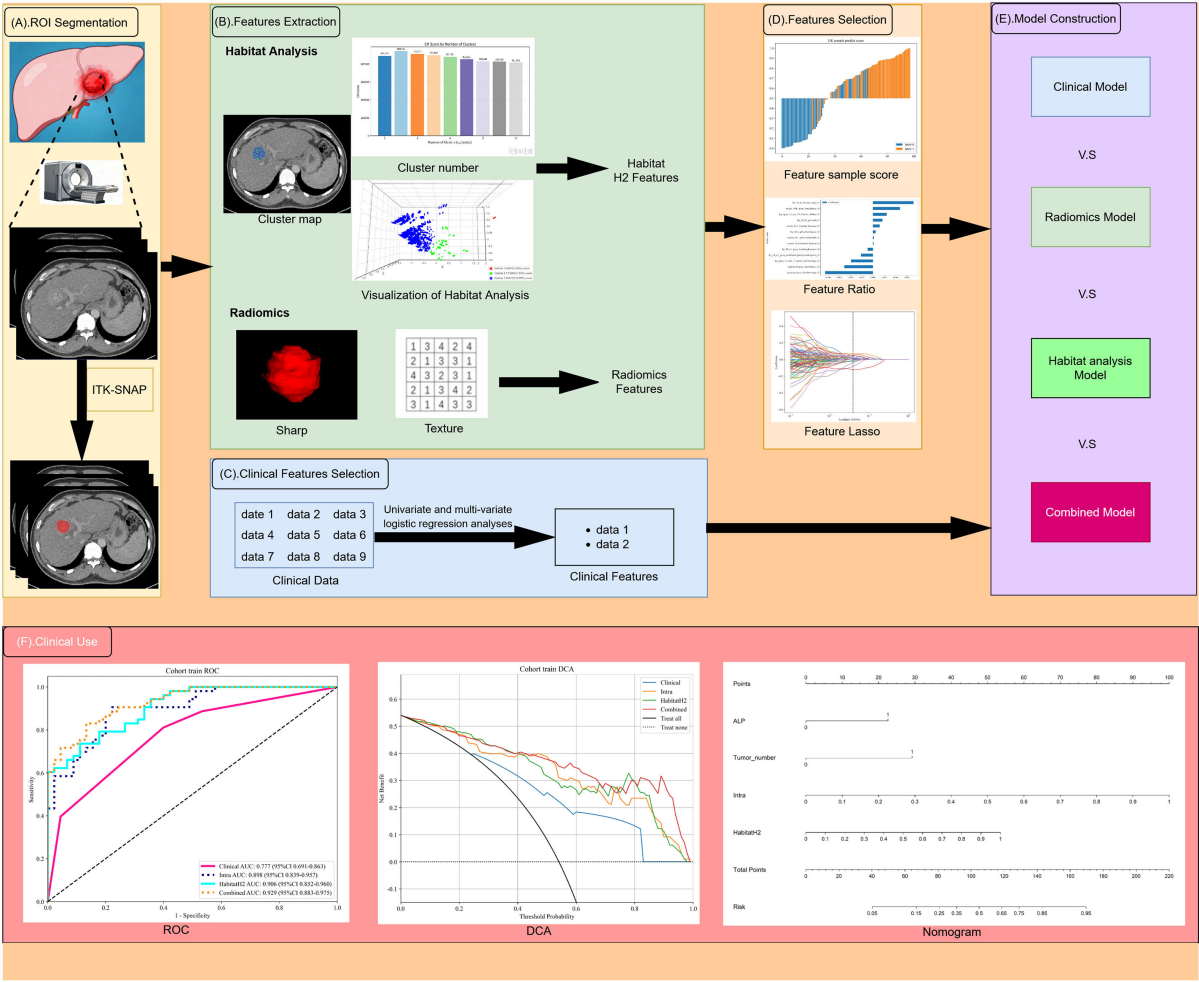
The workflow and rationale of the study.

Contrast-enhanced MRI (CE-MRI) is reported to predict microvascular invasion and recurrence-free survival after HCC resection (16, 17). Despite this, the clinical preference often leans towards contrast-enhanced CT due to its greater accessibility and cost-effectiveness. To date, no research has investigated the extraction of subregional radiomic features from the arterial phase of contrast-enhanced CT, nor has there been a comparison of the predictive performance of different subregions for early recurrence using various machine learning models. Additionally, the subregion most strongly associated with early recurrence remains unidentified, and there is a lack of integration of these findings with radiomic and clinical characteristics to create a visualized comprehensive model. This research aims to develop and validate a non-invasive model for predicting early recurrence after LT in HCC patients. This approach seeks to introduce innovative strategies for personalized treatment and clinical follow-up management in post-transplant patients. **Figure 1** provides a graphical representation of the study’s workflow and rationale, emphasizing the potential of this integrated model to enhance tumor recurrence prediction and improve the efficacy of follow-up strategies.

## Methods

### Study population and clinical data

**Figure 2:**
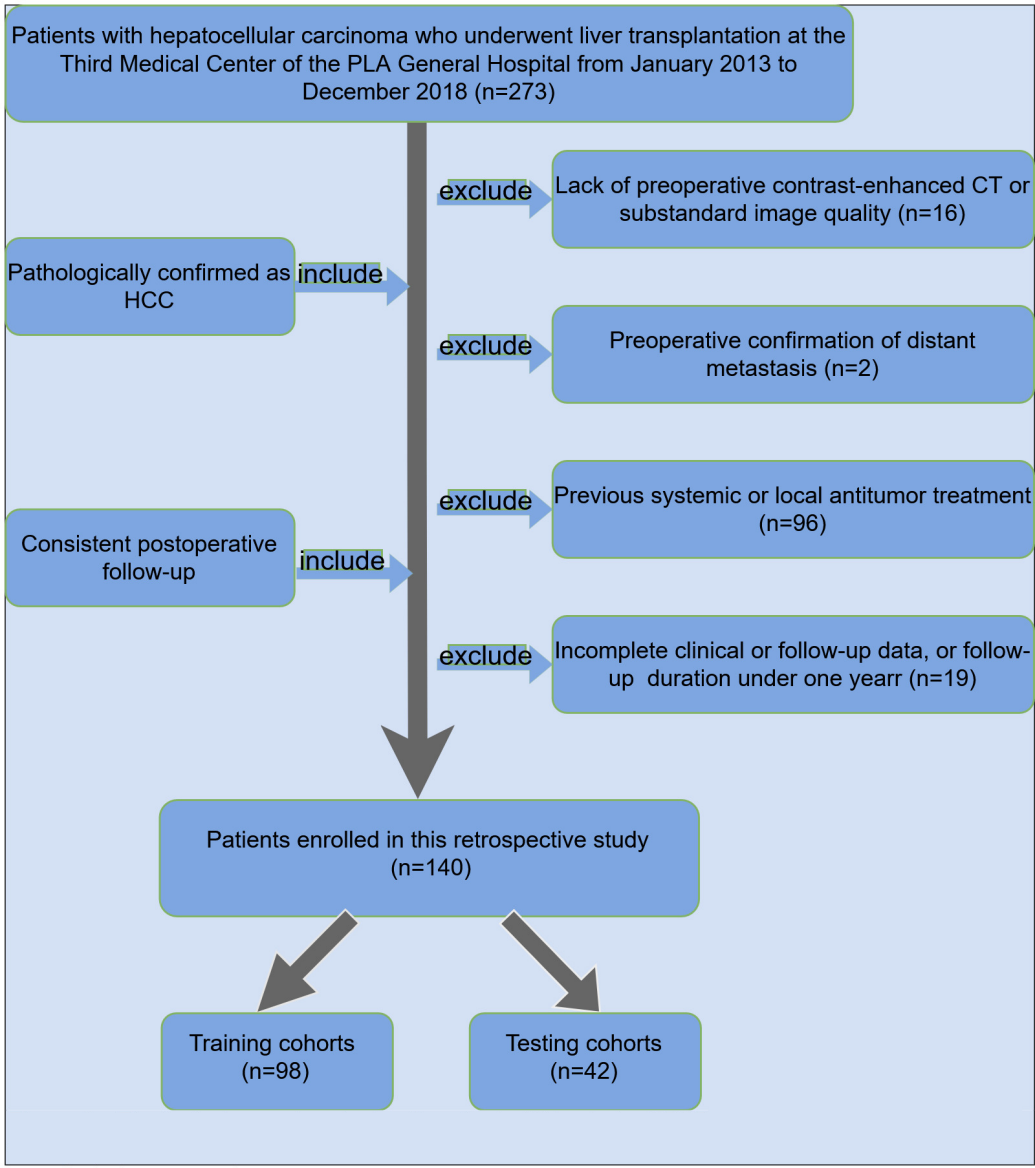
The inclusion and exclusion flowchart of this study. CECT, contrast-enhanced computed tomography, HCC, hepatocellular carcinoma.

The study complied with the Declaration of Helsinki (2013 revision) and was approved by the Medical Ethics Review Committee of the Third Medical Center, Chinese PLA General Hospital (Approval No. KY2024-016). Individual informed consent was not required due to the retrospective nature of the analysis. From January 2013 to December 2018, 273 patients with HCC who underwent liver transplantation were admitted to the Third Medical Center of the Chinese PLA General Hospital. Patients were randomly assigned to a training cohort and a testing cohort in a 7:3 ratio, following predefined inclusion and exclusion criteria. The inclusion criteria were: (I) pathologically confirmed HCC; and (II) consistent postoperative follow-up. Exclusion criteria were: (I) lack of preoperative contrast-enhanced CT or substandard image quality; (II) preoperative confirmation of distant metastasis; (III) previous systemic or local antitumor treatment; and (IV) incomplete clinical or follow-up data, or follow-up duration under one year. The study samples were divided into two groups: a training cohort containing 70% of the data and a testing cohort with the remaining 30%. **Figure 2** outlines the study population’s inclusion and exclusion criteria.

Preoperative clinical data for HCC patients were obtained from the hospital information management system, encompassing demographic details (age, sex), Child-Pugh liver function classification, and laboratory markers such as hepatitis B surface antigen (HBsAg), alpha-fetoprotein (AFP), and alkaline phosphatase (ALP). In addition, relevant data—such as whether patients met the Milan criteria—was collected. Postoperative pathological data, including Ki-67 expression, tumor count, and largest tumor diameter, were simultaneously gathered. Ki-67 expression was measured by calculating the percentage of cells that tested positive out of the total number of cells. Based on prior studies (18, 19), patients were categorized into low-expression (<10%) and high-expression (≥10%) groups after averaging the Ki-67 expression levels.

Postoperative outpatient follow-up for patients began one month after discharge. Follow-up assessments were conducted every three months. These evaluations included serological assessments such as liver function tests and serum alpha-fetoprotein levels, along with imaging examinations. The follow-up period continued until January 1, 2020, or ended upon tumor recurrence, loss to follow-up, or patient death. Early recurrence after liver transplantation for HCC is identified as the appearance of new tumors in the liver or at extrahepatic locations within one year post-surgery, confirmed by imaging or pathological analysis (20). At the same time, we recorded patients’ disease-free survival (DFS) during follow-up; this metric is calculated from the date of liver transplantation until the detection of recurrence.

### CECT image acquisition

An arterial phase contrast-enhanced CT (CECT) scan of the patient’s liver was performed using a 64-slice GE Healthcare Discovery CT750 HD scanner. The scan parameters included a detector collimation of 64×0.625 mm and a pitch of 1.375, with native slice thickness and spacing at 5 mm. The tube voltage was 120 kV, utilizing automatic tube current modulation. Images were reconstructed with a 1.25 mm slice thickness and interval. The patient was placed supine, and the scan covered the area from the diaphragmatic dome to the lower edge of the symphysis pubis in one breath-hold. A plain scan was initially conducted, followed by the intravenous injection of Omnipaque 320 contrast agent through the median cubital vein at 3.0 ml/s. Anterior-posterior enhanced CT images were acquired 25 seconds post-injection during the arterial phase using an anteroposterior scan. Post-extraction of sensitive data, the images were anonymized and saved in DICOM format.

### Tumor region of interest segmentation

We have established a voxel spacing standard to enhance the accuracy and uniformity of medical image processing. This protocol utilizes fixed-resolution resampling technology to ensure precise alignment across various ROIs. We standardized the resolution to enhance image quality for further analyses. Imaging parameters were set to a window level of 50 Hounsfield units and a window width of 450 Hounsfield units to improve image contrast and clarity for subsequent analyses.

Tumor ROIs were outlined using ITK-SNAP software (v3.8, www.itk-snap.org). When multiple liver tumors were identified, the largest tumor was chosen as the ROI. Each CT image was independently segmented on a slice-by-slice basis by two senior abdominal radiologists, each with over ten years of experience. Segmentation discrepancies were resolved in consultation with a radiology expert with 20 years of experience. Notably, none of the radiologists had access to clinical or pathological data during the process.

### Pixel-level characterization of local habitat features and zone generation

Precise localization of the tumor within the CT image is essential before generating the subregion. Each CT image is paired with a tumor mask of the same size, outlining the tumor region. Subsequently, each pixel within the tumor is considered a center point, and radiomic features are extracted by expanding one pixel in each of the up, down, left, right, front, and back directions. Pyradiomics (version 3.0.1) is utilized for this purpose. All features are normalized to a 0 to 1 range for clustering analysis.

Expanding the voxel count around the tumor and extracting more radiomic features enhances noise resistance, but it exponentially increases computational demands due to the necessity of local feature characterization for each tumor pixel. This study restricts radiomic features to 19, comprising three from first-order statistics, nine from the gray-level co-occurrence matrix (GLCM), three from the gray-level run length matrix (GLRLM), two from the gray-level size-zone matrix (GLSZM), and two from the neighboring gray-tone difference matrix (NGTDM). Most features were extracted from the GLCM, including metrics like Difference Entropy, Joint Entropy, Sum Entropy, Difference Variance, and Joint Energy. GLCM is a valuable tool for analyzing tumor image heterogeneity as it effectively captures subtle texture variations related to image irregularities and complexity (21). The local radiomic data for each pixel was converted into a multidimensional feature vector.

An unsupervised k-means clustering algorithm was utilized to create homogeneous subregions among tumor voxels, adjusting the clustering number (k) between 2 and 10 to control resolution. The optimal number of clusters was selected based on the Calinski-Harabasz (CH) index, using the maximum CH value as the criterion (22). In the clustering process, pixels in the same cluster were given the same color, creating a subregion map that visually represents the tumor’s distribution pattern.

### Feature extraction

Radiomics features were extracted from each patient’s AP-CECT images using Pyradiomics (version 3.0.1) across seven categories: first-order statistics, shape features, GLCM, GLSZM, GLRLM, NGTDM, and gray-tone dependent matrix (GLDM). Images were resampled to isotropic voxel spacing (1×1×1mm³) using cubic interpolation to maintain rotational invariance of texture features. From each region of interest, 1,834 radiomics features were extracted, including 360 first-order statistics, 14 shape features, 440 GLCM, 320 GLSZM, 320 GLRLM, 100 NGTDM, and 280 GLDM features.

### Feature selection and model building

To identify features with high reproducibility and low redundancy, an initial screening was conducted using independent samples t-tests, excluding features with P-values greater than 0.05.Pearson correlation coefficients were computed for the remaining highly reproducible features to evaluate their interrelationships, retaining only feature pairs with coefficients exceeding 0.9. In conclusion, the LASSO algorithm was utilized to determine non-zero coefficients with the optimal penalty parameter (λ), leading to the selection of independent and stable radiomic features. These filtered features were standardized through the Z-score method, wherein the mean and variance were computed for each feature column. Each feature column was standardized to a normal distribution by subtracting the mean and dividing by the standard deviation. Machine learning classification models were developed using the scikit-learn library after feature fusion and selection. The classification models comprised Logistic Regression (LR), Support Vector Machine (SVM), Extreme Random Tree (ExtraTree), and Extreme Gradient Boosting (XGBoost). Univariate and multivariate logistic regression analyses were performed for each clinical feature to identify independent risk factors for early recurrence after HCC liver transplantation. The most effective subregion’s machine learning classification model, identified through comparisons of subregional habitat analyses using the AP-CECT images, was chosen as the representative habitat analysis model. A comprehensive model was developed by integrating radiomic features, clinical characteristics, and habitat features. SHapley Additive exPlanations (SHAP) values were calculated for each prediction to clarify and interpret the decision-making processes of the combined model. The SHAP method, derived from game theory, measures each feature’s contribution to the probability of a specific model outcome.

### Statistical analysis

The Shapiro-Wilk test was employed to assess the normality of clinical characteristics. Continuous variables were analyzed using t-tests or Mann-Whitney U tests, depending on their distribution. The chi-square (χ²) test was utilized to analyze categorical variables. The model’s predictive performance was assessed using the area under the receiver operating characteristic curve (AUC), as well as accuracy, sensitivity, specificity, calibration curves, and decision curve analysis (DCA). Calibration curves assessed model calibration, with reliability verified by the Hosmer-Lemeshow goodness-of-fit test. DCA was used to evaluate the clinical utility of each predictive model, aiding in understanding their potential benefits in clinical settings. Data analyses were conducted using Python 3.7.12 on the OnekeyAI platform, version 4.9.1.Statistical analyses utilized Statsmodels v0.13.2, and radiomics feature extraction employed PyRadiomics v3.0.1.Machine learning algorithms such as LR, SVM, Extra Trees, and XGBoost were implemented using Scikit-learn version 1.0.2.

## Results

### Establishing a clinical model for HCC patients

Out of 140 eligible patients, 98 were assigned to the training cohort and 42 to the testing cohort, preserving a 7:3 ratio. **Table 1** summarizes the clinical characteristics of the patient groups. The cohorts exhibited similar baseline characteristics (P > 0.05). This study performed a detailed univariate and multi-variate logistic regression analysis on all clinical features. **Table 2** indicates that ALP levels and tumor number were key determinants in developing the clinical model, referred to as “Clinical” in subsequent figures and tables.

**Table 1:** The demographic and clinical-pathologic characteristics of patients.

Variable	Total (n = 140)	Training cohort (n = 98)	Testing cohort (n = 42)	p
AGE, Mean ± SD	50.44 ± 9.11	50.16 ± 9.67	51.07 ± 7.73	0.591
Gender, n (%)				1.000
Female	12 (8.57)	8 (8.16)	4 (9.52)	
Male	128 (91.43)	90 (91.84)	38 (90.48)	
AFP, ng/mL, n (%)				0.373
<200	99 (70.71)	72 (73.47)	27 (64.29)	
≥200	41 (29.29)	26 (26.53)	15 (35.71)	
ALP, U/L, n (%)				1.000
<135	93 (66.43)	65 (66.33)	28 (66.67)	
≥135	47 (33.57)	33 (33.67)	14 (33.33)	
HBsAg, n (%)				0.366
Negative	17 (12.14)	14 (14.29)	3 (7.14)	
Positive	123 (87.86)	84 (85.71)	39 (92.86)	
CHILD				0.553
A	63 (45.00)	42 (42.86)	21 (50.00)	
B	77 (55.00)	56 (57.14)	21 (50.00)	
Ki67, %, n (%)				0.094
<10%	80 (57.14)	61 (62.24)	19 (45.24)	
≥10%	60 (42.86)	37 (37.76)	23 (54.76)	
Largest tumor size, cm, n (%)				0.852
<5	80 (57.14)	55 (56.12)	25 (59.52)	
≥5	60 (42.86)	43 (43.88)	17 (40.48)	
Tumor number				0.395
Single	49 (35.00)	37 (37.76)	12 (28.57)	
Multiple	91 (65.00)	61 (62.24)	30 (71.43)	
Recurrence, n (%)				0.954
Yes	79 (56.43)	53 (67.09)	26 (32.91)	
No	61 (43.57)	45 (73.77)	16 (26.23)	
DFS, Mean ± SD	28.74 ± 25.41	29.21 ± 24.93	27.62 ± 26.75	0.438
Milan criteria				0.398
In	72 (51.43)	48 (48.98)	24 (57.14)	
Out	68 (48.57)	50 (51.02)	18 (42.86)	

AFP, alpha-fetoprotein; ALP, alkaline phosphatase; DFS, disease-free survival.

**Table 2:** Univariate and multi-variate logistic regression analyses for patients in the training cohort.

Variables	Univariate analysis		Multi-variable analysis	
	P	HR (95%CI)	P	HR (95%CI)
Age, years	0.525	1.003 (0.996 - 1.009)		
Gender (male vs female)	0.527	1.143 (0.807 - 1.618)		
HBsAg (negative vs positive)	0.383	1.211 (0.844 – 1.737)		
Child-Pugh class (A vs B)	0.424	1.240 (0.797 - 1.929)		
AFP, ng/mL (≥200 vs <200)	0.004	4.200 (1.853 – 9.526)	0.315	2.642 (0.538 – 12.96)
ALP, U/L (≥135 vs <135)	0.005	3.125 (1.602 - 6.098)	0.012	6.134 (1.870 – 20.11)
Largest tumor size, cm (<5 vs ≥5)	0.051	1.867 (1.103 – 3.158)		
Tumor number (single vs multiple)	0.002	2.389 (1.505 – 3.792)	<0.05	9.302 (3.414 – 25.33)
Ki67, % (≥10 vs <10)	0.017	2.364(1.309 – 4.272)	0.863	1.107 (0.421 - 2.907)

AFP, alpha-fetoprotein; ALP, alkaline phosphatase.

### Establishing a radiomics model for HCC patients

We extracted 1,834 feature parameters from the analysis of AP-CECT images. By employing Lasso dimensionality reduction and cross-validation, we identified twenty radiomic features significantly linked to early recurrence after liver transplantation in HCC. Subsequently, based on the selected features and their corresponding weights, we computed a Radiomics Score (Rad-score) for each patient using a specified formula and developed a radiomics model, referred to as “Intra” in the subsequent figures and tables.Rad-score= 0.5408163265306122 -0.081024 * intra_exponential_glrlm_RunVariance –0.022597 * intra_exponential_glszm_LargeAreaLowGrayLevelEmphasis +0.054244 * intra_lbp_3D_k_firstorder_Range +0.015418 * intra_lbp_3D_k_glszm_SizeZoneNonUniformity +0.008718 * intra_lbp_3D_m1_glszm_ZoneEntropy –0.040274 * intra_lbp_3D_m2_glrlm_ShortRunLowGrayLevelEmphasis +0.000346 * intra_lbp_3D_m2_glszm_LowGrayLevelZone Emphasis –0.009998 * intra_lbp_3D_m2_glszm_SizeZoneNonUniformity Normalized +0.071650 * intra_lbp_3D_m2_glszm_ZoneEntropy +0.002960 * intra_log_sigma_1_0_mm_3D_glcm_Idm +0.042704 * intra_log_sigma_3_0_mm_3D_firstorder_Root MeanSquared –0.124385 * intra_squareroot_glszm_ZonePercentage +0.026970 * intra_wavelet_HHH_firstorder_Kurtosis +0.006147 * intra_wavelet_HHH_firstorder_Maximum +0.033100 * intra_wavelet_HHL_firstorder_Mean +0.014897 * intra_wavelet_HHL_firstorder_RootMeanSquared +0.008075 * intra_wavelet_LLH_firstorder_Median –0.019563 * intra_wavelet_LLH_firstorder_Skewness +0.047902 * intra_wavelet_LLL_firstorder_Skewness +0.017134 * intra_wavelet_LLL_glcm_ClusterShade

### Establishing a habitat analysis model for HCC patients

Through the analysis of AP-CECT images and the comparison of CH values using k-means clustering, it was determined that the highest CH values were observed when utilizing three distinct habitat subregions, as detailed in **Table 3**. Consequently, the resulting habitat models were designated as HabitatH1, HabitatH2, and HabitatH3. The HabitatH2 sub-region was chosen for habitat analysis model construction after analyzing the performance parameters in **Table 4**. The habitat analysis model is referred to as “HabitatH2” in subsequent figures and tables.

**Table 3:** CH_score of different clusters.

n_clusters	CH_score
2	892298.3846938999
3	944735.020893107
4	913512.4848723729
5	899506.5348190662
6	881331.4821779553
7	853515.1878161698
8	830343.3217142309
9	828319.2064803365
10	817160.8216012297

CH, Calinski-Harabasz.

**Table 4:** Metrics in the training and testing cohorts for predicting early recurrence across habitat subregions.

Habitat	Accuracy	AUC	95% CI	Sensitivity	Specificity	PPV	NPV	Recall	Cohort
HabitatH1	0.867	0.920	0.866 - 0.974	0.887	0.844	0.870	0.864	0.887	Training
HabitatH2	0.806	0.906	0.852 - 0.960	0.736	0.889	0.886	0.741	0.736	Training
HabitatH3	0.816	0.864	0.792 - 0.936	0.830	0.800	0.830	0.800	0.830	Training
HabitatH1	0.738	0.733	0.575 - 0.892	0.846	0.562	0.759	0.692	0.846	Testing
HabitatH2	0.810	0.817	0.681 - 0.954	0.885	0.687	0.821	0.786	0.885	Testing
HabitatH3	0.762	0.779	0.615 - 0.943	0.769	0.750	0.833	0.667	0.769	Testing

### Performance comparison of prediction models

The optimal machine learning models identified within the clinical model were LR, Extra Trees, and XGBoost, all of which demonstrated consistent performance. This consistency is likely due to the limited number of features, specifically two, employed in the clinical model’s construction, as elaborated in **Supplementary File S1**. The training cohort achieved accuracy, sensitivity, specificity, and AUC of 0.714, 0.811, 0.600, and 0.777 (95% CI: 0.691–0.863), respectively. The accuracy, sensitivity, specificity, and AUC of the testing cohort were 0.810, 0.923, 0.625, and 0.815, respectively (95% CI: 0.685–0.945).

The optimal machine learning model in the radiomics model was LR, as detailed in **Supplementary File S2**. The accuracy, sensitivity, specificity, and AUC of the training cohort were 0.847, 0.906, 0.778, and 0.898, respectively (95% CI: 0.839–0.957). The accuracy, sensitivity, specificity, and AUC of the testing cohort were 0.738, 0.731, 0.750, and 0.796, respectively (95% CI: 0.657–0.934).

The optimal machine learning model in the habitat analysis model constructed by HabitatH2 was LR, as detailed in **Supplementary File S3**. The accuracy, sensitivity, specificity, and AUC of the training cohort were 0.806, 0.736, 0.889, and 0.906 (95% CI: 0.852–0.960), respectively. while the testing cohort yielded accuracy, sensitivity, specificity, and AUC of 0.810, 0.885, 0.687, and 0.817 (95% CI: 0.681–0.954), respectively.

A combined model integrating clinical, radiomic, and habitat features achieved the training cohort accuracy, sensitivity, specificity, and AUC of 0.847, 0.830, 0.867, and 0.929 (95% CI: 0.883–0.975), respectively. The accuracy, sensitivity, specificity, and AUC of the testing cohort were 0.833, 0.731, 1.000, and 0.882 (95% CI: 0.783–0.982), respectively. The combined model is referred to as “Combined” in subsequent figures and tables.

**Figure 3:**
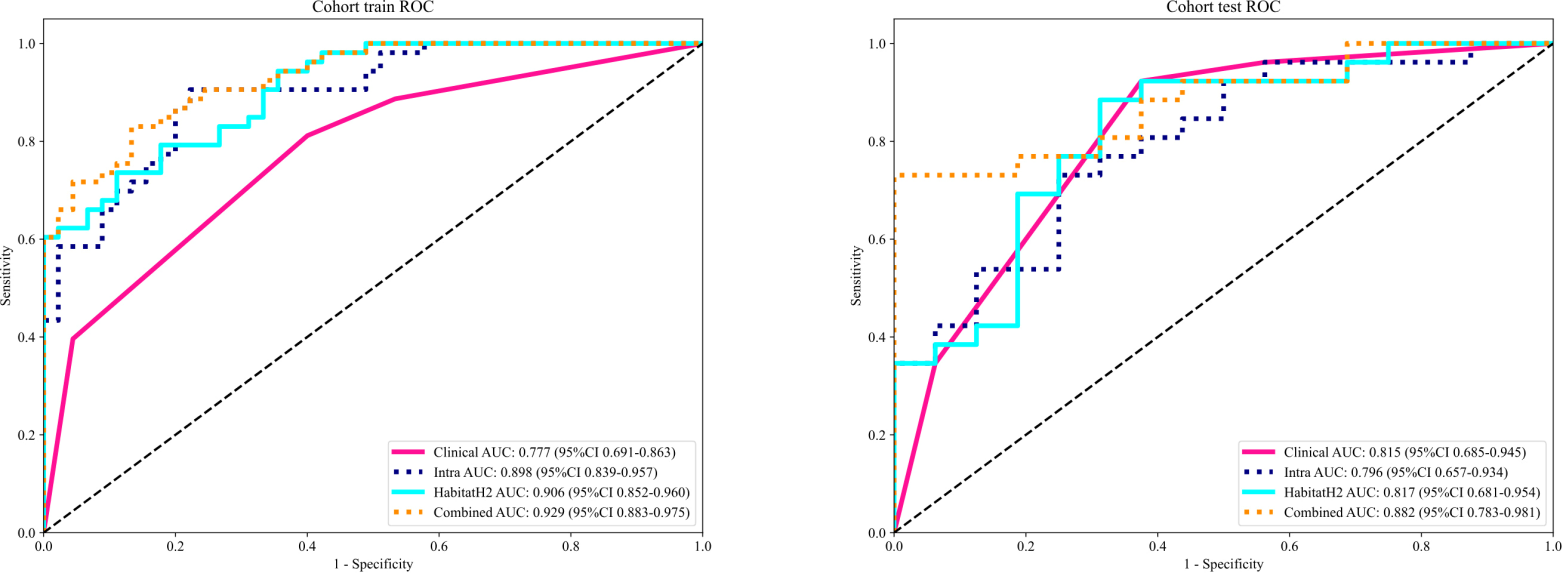
The ROC curves in the training and testing cohorts.

**Figure 4:**
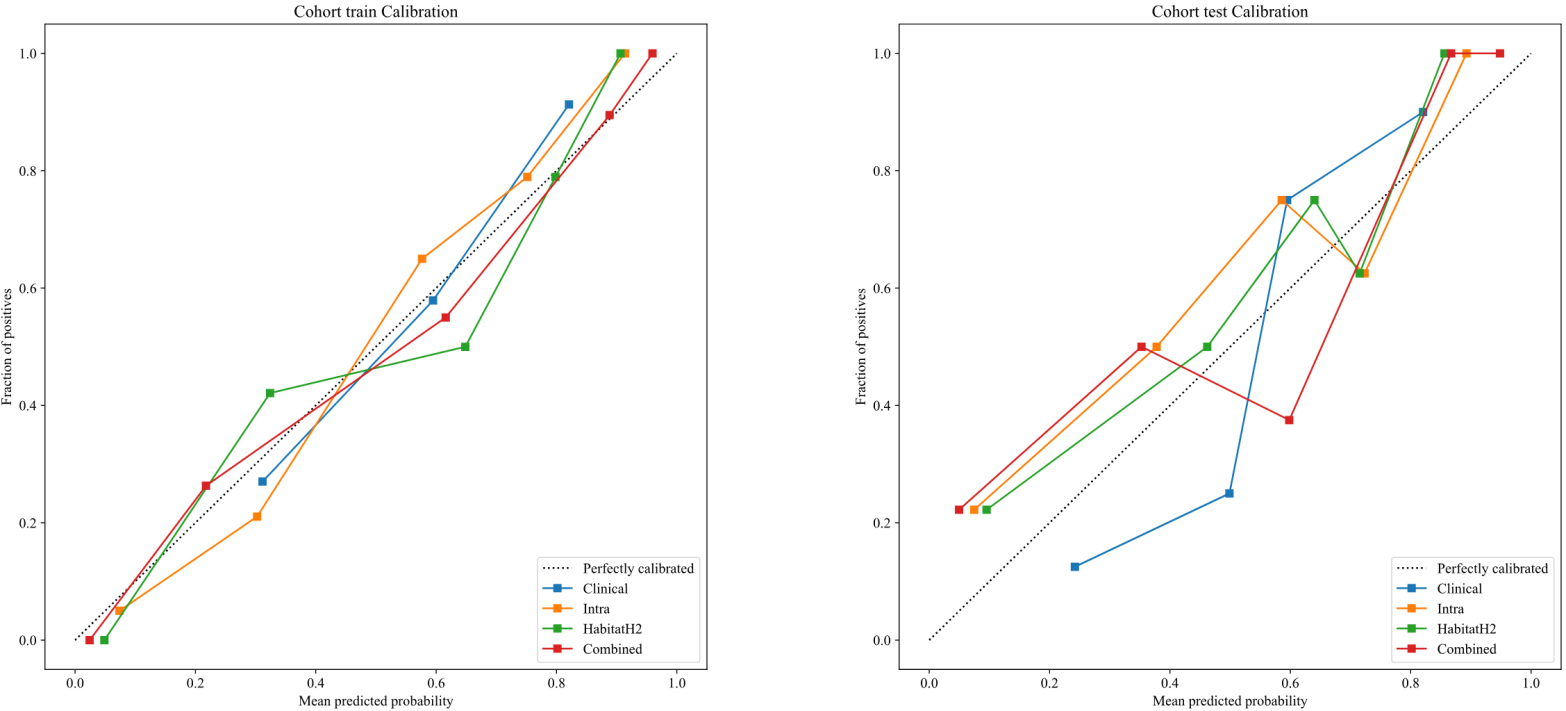
The calibration curves of different models in the training and testing cohorts.

**Figure 5:**
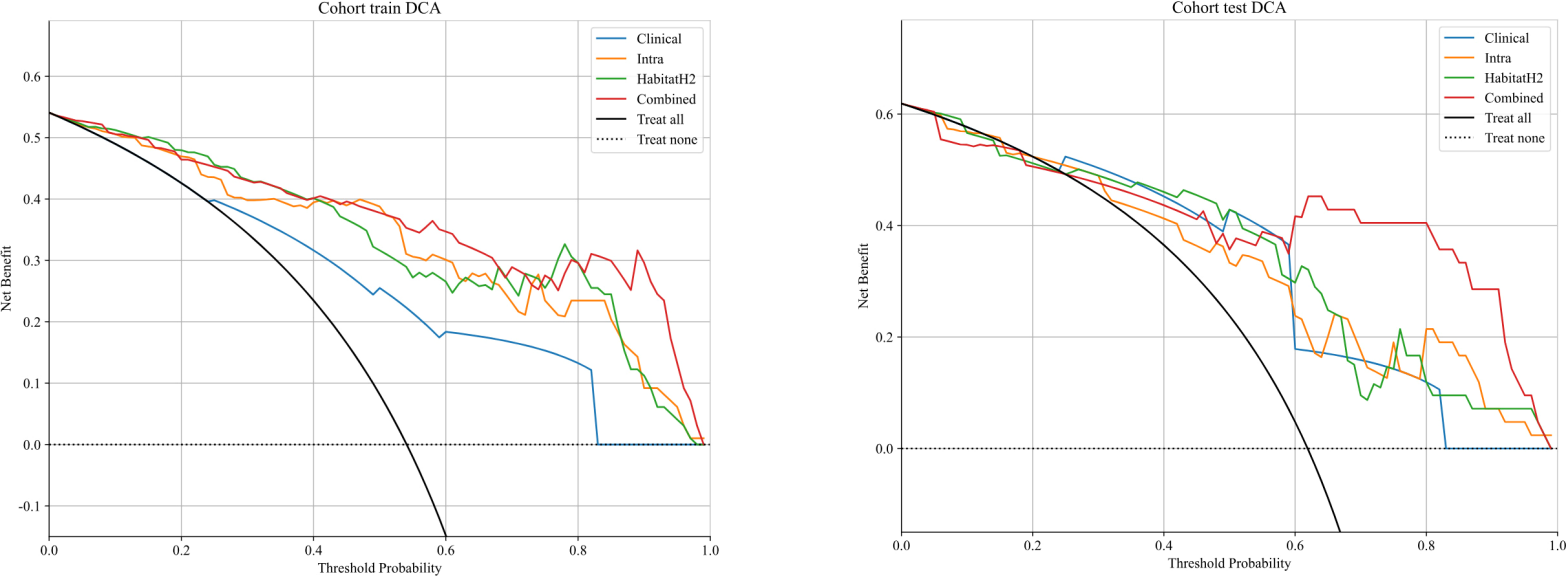
The DCA of different models in the training and testing cohorts.

**Figure 6:**
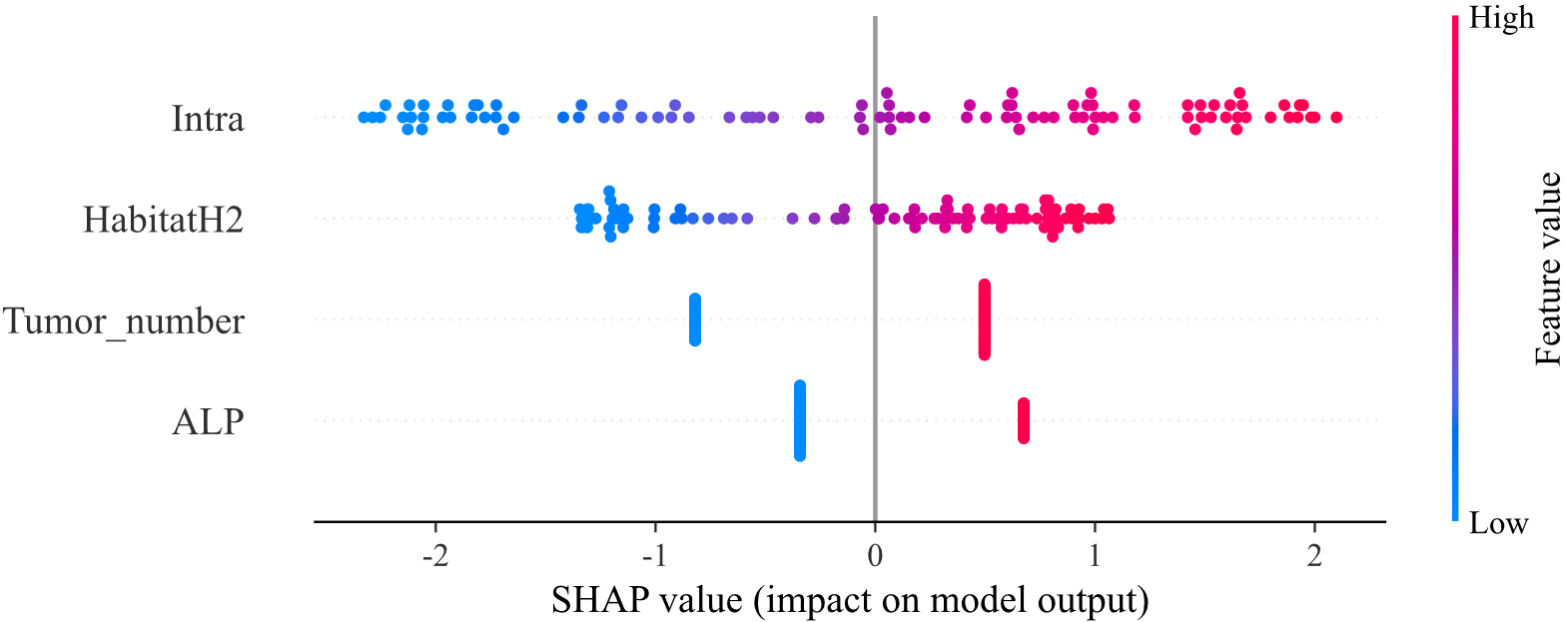
SHAP summary plots of the combined model.

**Figure 7:**
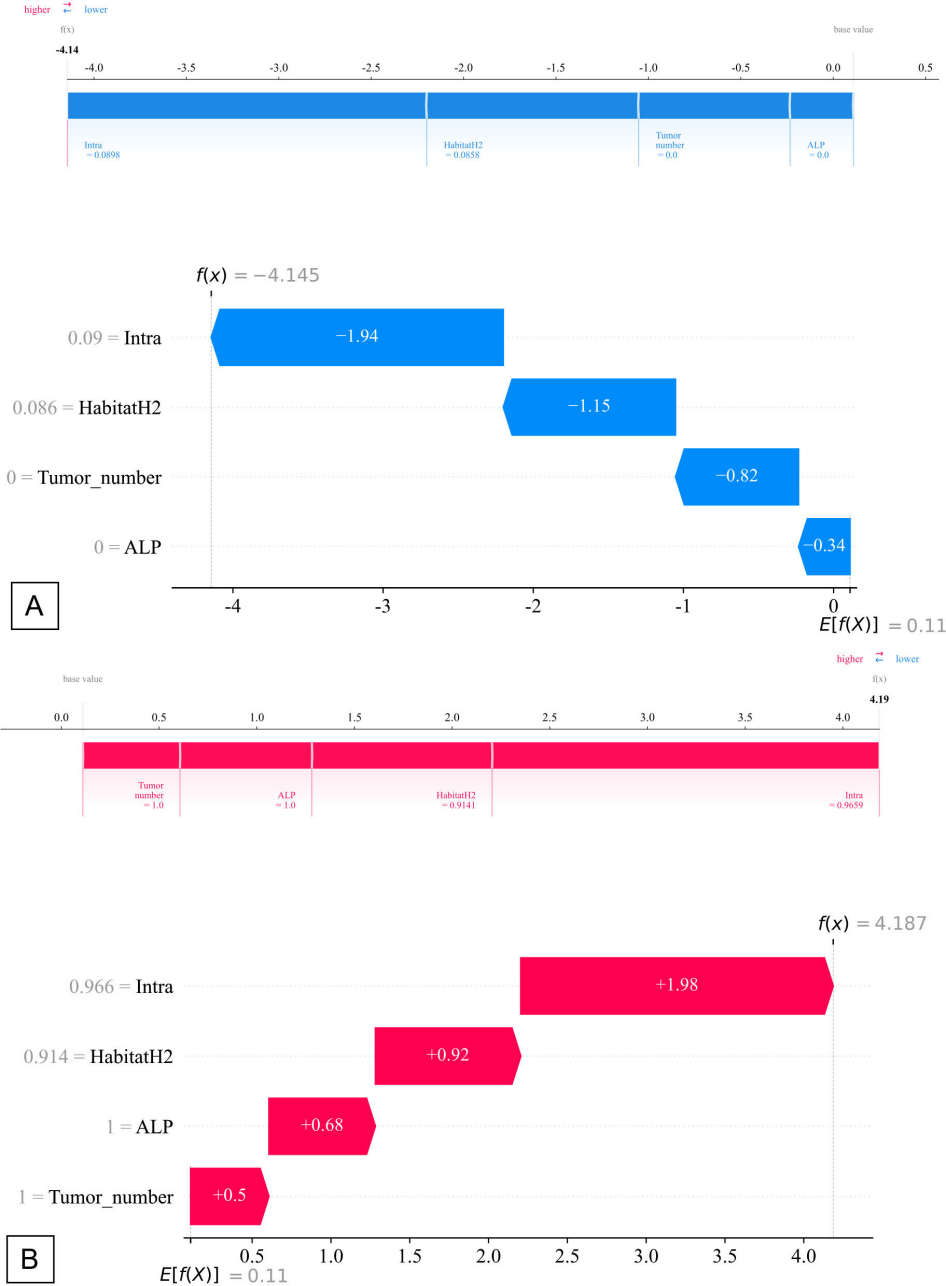
SHAP force plots explains how the combined model distinguishes between patients who experience early recurrence and those who do not. Panel **(A)** denotes the patient without early recurrence, while panel **(B)** denotes the patient with early recurrence.

**Figure 8:**
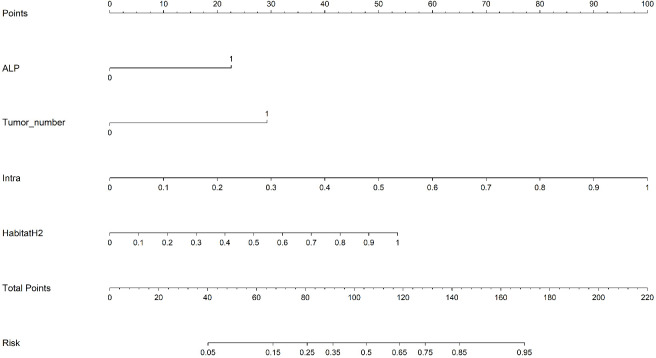
The nomogram for the combined model.

The combined model emerged as the optimal choice due to its superior accuracy, sensitivity, specificity, and AUC compared to other models (**Table 5**; **Figure 3**). The calibration curves demonstrate that the combined model’s predictions align more accurately with actual outcomes in both training and testing cohorts (**Figure 4**). Moreover, DCA across these cohorts demonstrates that the combined model provides the highest clinical net benefit at most threshold probabilities (**Figure 5**). SHAP value analysis identifies radiological and habitat features as the primary risk factors in the combined model (**Figure 6**). SHAP force plots explains how the combined model distinguishes between patients who experience early recurrence and those who do not (**Figure 7**). Each prediction begins from a uniform baseline value, with arrow colors representing positive (red) or negative (blue) contributions. We created a combined model that integrates radiomics and habitat analysis, using nomogram visualization to predict the risk of early recurrence after LT for HCC (**Figure 8**).

**Table 5:** Metrics in the training and testing cohorts for predicting early recurrence across different models.

Model	Accuracy	AUC	95% CI	Sensitivity	Specificity	PPV	NPV	Recall	Cohort
Clinical	0.714	0.777	0.691 - 0.863	0.811	0.600	0.705	0.730	0.811	Training
Intra	0.847	0.898	0.839 - 0.957	0.906	0.778	0.828	0.875	0.906	Training
HabitatH2	0.806	0.906	0.852 - 0.960	0.736	0.889	0.886	0.741	0.736	Training
Combined	0.847	0.929	0.883 - 0.975	0.830	0.867	0.880	0.812	0.830	Training
Clinical	0.810	0.815	0.685 - 0.945	0.923	0.625	0.800	0.833	0.93	Testing
Intra	0.738	0.796	0.657 - 0.934	0.731	0.750	0.826	0.632	0.731	Testing
HabitatH2	0.810	0.817	0.681 - 0.954	0.885	0.687	0.821	0.786	0.885	Testing
Combined	0.833	0.882	0.783 - 0.982	0.731	1.000	1.000	0.696	0.731	Testing

## Discussion

Early recurrence is a critical determinant of HCC prognosis (23). LT, as a key treatment for HCC, would be more conducive to optimizing organ resource allocation and improving long-term prognosis if high-risk patients for postoperative recurrence could be identified during preoperative assessment (24). Gu et al. (25) indicated that up to 30% of high-risk postoperative patients experience distant metastases, with extrahepatic recurrence rates as high as 97.7%. Consequently, implementing more aggressive interventions for individuals at high risk of early recurrence following HCC liver transplantation can optimize personalized treatment strategies and postoperative follow-up monitoring plans, ultimately improving patient survival rates.

Recent research has focused on early postoperative HCC recurrence through CT imaging, examining the relationship between recurrence and radiomic features, yielding promising predictive results (26–28). Most research to date has predominantly focused on radiomic features derived from the entire tumor region. This comprehensive approach may introduce confounding factors like hemorrhage, necrosis, cystic changes, and edema, potentially obscuring heterogeneous expression patterns and diminishing model predictive performance. Habitat analysis, a novel technique, overcomes this limitation by focusing on subregional histological analysis within tumors, enabling more accurate quantification of tumor subregions linked to growth or invasiveness (29). This method elucidates spatial tumor heterogeneity, distinguishing it from traditional whole-tumor radiomics analysis. Similarly, Li et al. (11) demonstrated that CT-based habitat models effectively predict tumor phenotypes and prognosis in non-small cell lung cancer.

In this study, we objectively extracted tumor subregions using features from various statistical methods: three from first-order statistics, nine from the GLCM, three from the GLRLM, two from the GLSZM, and two from the NGTDM. The pixels were clustered according to their local features, identifying three distinct subregions. The GLCM features constituted the predominant proportion due to their efficacy in capturing subtle texture variations associated with image irregularities and complexity, which is essential for the analysis of tumor heterogeneity (21). We developed a combined model that merges radiomics with habitat analysis, significantly improving predictive performance over individual models. The combined model demonstrated excellent predictive performance, achieving AUC values of 0.929 (95% CI 0.883–0.975) in the training cohort and 0.882 (95% CI 0.783–0.982) in the testing cohort. Moreover, the calibration curve for the combined model demonstrated strong concordance with actual outcomes. DCA indicated that the combined model possesses substantial clinical utility for predicting early recurrence following HCC liver transplantation. Our study provides important insights for HCC management.

The SHAP method assesses each feature’s unique contribution to a model’s prediction of observed outcomes (30). This assessment significantly improves the interpretability of machine learning models (31). The study indicates that radiomics features from the arterial phase and microenvironment characteristics are the primary risk factors, suggesting a connection between HCC hemodynamics, microenvironment, and aggressive biological behavior (32), with early recurrence possibly due to tumor invasiveness. The radiomic and habitat features primarily derive from wavelet features, which proficiently capture heterogeneity across various spatial scales (15, 33).

ITH, observable as local or global variations on CT images, may arise from differences in tumor cell composition or properties. These habitat features offer detailed insights into HCC aggressiveness and enhance the quantification of tumor subregions linked to growth or invasiveness (29). This research highlights the importance of using habitat analysis methods to describe the complex tumor microenvironment. Invasive subregions are crucial for prognosis and treatment response in medicine.

Our research utilized an integrated approach that combines radiomics and habitat analysis with clinical characteristics, resulting in a model with superior predictive performance. Despite the promising nature of our findings, several limitations must be acknowledged. Firstly, as a single-center retrospective study, there is a need for multicenter participation to validate the clinical applicability and reliability of the combined model. Secondly, the lack of precise alignment between CT image habitat subregions and pathological specimens underscores the need for more extensive prospective studies to investigate the biological behavior within each subregion. This study did not explore the relationships between genomics, radiomics, and habitat analysis. Future research should include more genetic data dimensions to improve the model’s predictive power.

## Conclusion

In summary, habitat analysis enables the quantification and visualization of tumor subregions, providing crucial insights for predicting early recurrence after liver transplantation for HCC. This study successfully developed a combined model based on radiomics and habitat analysis, which effectively predicts early postoperative recurrence in a preoperative setting. This advancement provides more precise guidance for the formulation of clinical treatment and follow-up monitoring strategies.

## Data Availability

The original contributions presented in the study are included in the article/**Supplementary Material**. Further inquiries can be directed to the corresponding authors.
