# Supplementary material for: A combined radiomics and habitat analysis model for predicting early recurrence of HCC after liver transplantation
Source: Front Oncol. 2026 May 26;16:1789990. doi: 10.3389/fonc.2026.1789990 (PMC13246378; doi:10.3389/fonc.2026.1789990)
Supplement: Supplementary file 2 [file Table1.docx]

| **Model** | **Accuracy** | **AUC** | **95% CI** | **Sensitivity** | **Specificity** | **PPV** | **NPV** | **Recall** | **Cohort** |
| --- | --- | --- | --- | --- | --- | --- | --- | --- | --- |
| LR | 0.714 | 0.777 | 0.691 - 0.863 | 0.811 | 0.600 | 0.705 | 0.730 | 0.811 | Training |
| SVM | 0.714 | 0.655 | 0.543 - 0.766 | 0.811 | 0.600 | 0.705 | 0.730 | 0.811 | Training |
| ExtraTree | 0.714 | 0.777 | 0.691 - 0.863 | 0.811 | 0.600 | 0.705 | 0.730 | 0.811 | Training |
| XGBoost | 0.714 | 0.777 | 0.691 - 0.863 | 0.811 | 0.600 | 0.705 | 0.730 | 0.811 | Training |
| LR | 0.810 | 0.815 | 0.685 - 0.945 | 0.923 | 0.625 | 0.800 | 0.833 | 0.923 | Testing |
| SVM | 0.810 | 0.743 | 0.576 - 0.910 | 0.923 | 0.625 | 0.800 | 0.833 | 0.923 | Testing |
| ExtraTree | 0.810 | 0.815 | 0.685 - 0.945 | 0.923 | 0.625 | 0.800 | 0.833 | 0.923 | Testing |
| XGBoost | 0.810 | 0.815 | 0.685 - 0.945 | 0.923 | 0.625 | 0.800 | 0.833 | 0.923 | Testing |

**Table S1**: Comparison of performance metrics for predicting early recurrence among different machine learning models in clinical models across the training and testing cohorts.
