# Supplementary material for: A combined radiomics and habitat analysis model for predicting early recurrence of HCC after liver transplantation
Source: Front Oncol. 2026 May 26;16:1789990. doi: 10.3389/fonc.2026.1789990 (PMC13246378; doi:10.3389/fonc.2026.1789990)
Supplement: Supplementary file 3 [file Table2.docx]

| **Model** | **Accuracy** | **AUC** | **95% CI** | **Sensitivity** | **Specificity** | **PPV** | **NPV** | **Recall** | **Cohort** |
| --- | --- | --- | --- | --- | --- | --- | --- | --- | --- |
| LR | 0.847 | 0.898 | 0.839 - 0.957 | 0.906 | 0.778 | 0.828 | 0.875 | 0.906 | Training |
| SVM | 0.735 | 0.732 | 0.630 - 0.834 | 0.717 | 0.756 | 0.776 | 0.694 | 0.717 | Training |
| ExtraTree | 0.755 | 0.819 | 0.739 - 0.898 | 0.830 | 0.667 | 0.746 | 0.769 | 0.830 | Training |
| XGBoost | 0.776 | 0.762 | 0.682 - 0.843 | 0.925 | 0.600 | 0.731 | 0.871 | 0.925 | Training |
| LR | 0.738 | 0.796 | 0.657 - 0.934 | 0.731 | 0.750 | 0.826 | 0.632 | 0.731 | Testing |
| SVM | 0.619 | 0.632 | 0.457 - 0.807 | 0.538 | 0.750 | 0.778 | 0.500 | 0.538 | Testing |
| ExtraTree | 0.667 | 0.696 | 0.531 - 0.861 | 0.577 | 0.812 | 0.833 | 0.542 | 0.577 | Testing |
| XGBoost | 0.690 | 0.666 | 0.516 - 0.816 | 0.769 | 0.562 | 0.741 | 0.600 | 0.769 | Testing |

**Table S2**: Comparison of performance metrics for predicting early recurrence among different machine learning models in radiomics models across the training and testing cohorts.
