# Supplementary material for: A combined radiomics and habitat analysis model for predicting early recurrence of HCC after liver transplantation
Source: Front Oncol. 2026 May 26;16:1789990. doi: 10.3389/fonc.2026.1789990 (PMC13246378; doi:10.3389/fonc.2026.1789990)
Supplement: Supplementary file 4 [file Table3.docx]

| **Model** | **Accuracy** | **AUC** | **95% CI** | **Sensitivity** | **Specificity** | **PPV** | **NPV** | **Recall** | **Cohort** |
| --- | --- | --- | --- | --- | --- | --- | --- | --- | --- |
| LR | 0.806 | 0.906 | 0.852 - 0.960 | 0.736 | 0.889 | 0.886 | 0.741 | 0.736 | Training |
| SVM | 0.837 | 0.886 | 0.819 - 0.953 | 0.943 | 0.711 | 0.794 | 0.914 | 0.943 | Training |
| ExtraTree | 0.786 | 0.855 | 0.783 - 0.928 | 0.887 | 0.667 | 0.758 | 0.833 | 0.887 | Training |
| XGBoost | 0.847 | 0.886 | 0.818 - 0.953 | 0.887 | 0.800 | 0.839 | 0.857 | 0.887 | Training |
| LR | 0.810 | 0.817 | 0.681 - 0.954 | 0.885 | 0.687 | 0.821 | 0.786 | 0.885 | Testing |
| SVM | 0.690 | 0.786 | 0.649 - 0.923 | 0.538 | 0.937 | 0.933 | 0.556 | 0.538 | Testing |
| ExtraTree | 0.690 | 0.770 | 0.627 - 0.914 | 0.654 | 0.750 | 0.810 | 0.571 | 0.654 | Testing |
| XGBoost | 0.738 | 0.750 | 0.594 - 0.906 | 0.731 | 0.750 | 0.826 | 0.632 | 0.731 | Testing |

**Table S3**: Comparison of performance metrics for predicting early recurrence among different machine learning models in HabitatH2 across the training and testing cohorts.
